# Robust identification of temporal biomarkers in longitudinal omics studies

**DOI:** 10.1093/bioinformatics/btac403

**Published:** 2022-06-28

**Authors:** Ahmed A Metwally, Tom Zhang, Si Wu, Ryan Kellogg, Wenyu Zhou, Kevin Contrepois, Hua Tang, Michael Snyder

**Affiliations:** Department of Genetics, Stanford University, Stanford, CA 94305, USA; Illumina Artificial Intelligence Laboratory, Illumina Inc., San Diego, CA 92122, USA; Systems and Biomedical Engineering Department, Faculty of Engineering, Cairo University, Giza 12613, Egypt; Department of Computer Science, Columbia University, New York, NY 10027, USA; Department of Genetics, Stanford University, Stanford, CA 94305, USA; Department of Genetics, Stanford University, Stanford, CA 94305, USA; Department of Bioengineering, Stanford University, Stanford, CA 94305, USA; Department of Genetics, Stanford University, Stanford, CA 94305, USA; Department of Genetics, Stanford University, Stanford, CA 94305, USA; Department of Genetics, Stanford University, Stanford, CA 94305, USA; Department of Genetics, Stanford University, Stanford, CA 94305, USA

## Abstract

**Motivation:**

Longitudinal studies increasingly collect rich ‘omics’ data sampled frequently over time and across large cohorts to capture dynamic health fluctuations and disease transitions. However, the generation of longitudinal omics data has preceded the development of analysis tools that can efficiently extract insights from such data. In particular, there is a need for statistical frameworks that can identify not only which omics features are differentially regulated between groups but also over what time intervals. Additionally, longitudinal omics data may have inconsistencies, including non-uniform sampling intervals, missing data points, subject dropout and differing numbers of samples per subject.

**Results:**

In this work, we developed *OmicsLonDA*, a statistical method that provides robust identification of time intervals of temporal omics biomarkers. *OmicsLonDA* is based on a semi-parametric approach, in which we use smoothing splines to model longitudinal data and infer significant time intervals of omics features based on an empirical distribution constructed through a permutation procedure. We benchmarked *OmicsLonDA* on five simulated datasets with diverse temporal patterns, and the method showed specificity greater than 0.99 and sensitivity greater than 0.87. Applying *OmicsLonDA* to the iPOP cohort revealed temporal patterns of genes, proteins, metabolites and microbes that are differentially regulated in male versus female subjects following a respiratory infection. In addition, we applied *OmicsLonDA* to a longitudinal multi-omics dataset of pregnant women with and without preeclampsia, and *OmicsLonDA* identified potential lipid markers that are temporally significantly different between the two groups.

**Availability and implementation:**

We provide an open-source R package (https://bioconductor.org/packages/OmicsLonDA), to enable widespread use.

**Supplementary information:**

Supplementary data are available at *Bioinformatics* online.

## Introduction

Human health is highly dynamic, and there is great interest in better understanding how wellness and disease states fluctuate over time in relation to different variables such as lifestyle or treatment perturbations. While genetics provides a blueprint for life, health states are also reflected by many other ‘omics’ such as transcriptomics, proteomics, metabolomics, lipidomics, and microbiomics. With rapid advances and decreasing costs in sequencing and mass spectrometry, many studies are beginning to measure comprehensive omics profiles at frequent timepoints across many individuals. Longitudinal omics studies generate enormous datasets; however, there is currently a major bottleneck in analyzing these data to extract and interpret meaningful findings. In particular, there is a need for robust statistical methods for longitudinal omics.

Longitudinal omics data have their own properties that differentiate them from cross-sectional experiments, including high dimensional feature space, temporal and intrapersonal variation, and samples characterized by heterogeneity of various natures. These heterogeneities include a different number of samples per subject, uncaptured data points, variable time of sample collection ‘sampled non-uniformly’ and omics features often represent a biological process that usually exhibits temporal variation. Another aspect is the variability in temporal dependence structure, ‘variance–covariance structure’, between repeated measurements. All of these characteristics of longitudinal omics data make the analysis a challenging task. Methods developed for longitudinal omics data analysis can be categorized into the following groups: (i) methods that extract omics biomarkers for a specific phenotype (Storey *et al.*, 2005), (ii) methods that build mechanistic models to describe the underlying mechanism involved in gene regulation, metabolism or protein–protein interaction causally related to specific phenotype (Bordbar *et al.*, 2011; Gonçalves *et al.*, 2013; Mardinoglu *et al.*, 2013) and (iii) identifying clusters of omics features that have similar expression patterns (Bar-Joseph, 2004).

For the class of methods that identify omics biomarkers, many statistical models have been proposed. The joint mixed model, which is widely used, links separate linear mixed models by allowing their model-specific random effects to be correlated (Verbeke *et al.*, 2014). The advantages of this approach include well-established theory and efficiency gains (Gueorguieva and Sanacora, 2006; Molenberghs and Verbeke, 2000). More importantly, a joint random-effect model allows the correlation between different outcomes to be assessed. It can provide a succinct summary of not only how the evolution of one outcome variable is correlated to the evolution of another outcome but also how the correlation between outcomes changes over time ('evolution of association') (Fieuws and Verbeke, 2004). On the other hand, the mixed-effect model comes with its set of assumptions, such as homogeneity of variance of the residuals being equal across groups (Wang *et al.*, 2017) and normality of the residuals (Santos Nobre and da Motta Singer, 2007). In many situations, these assumptions are violated. With the rapidly increasing size and complexity of omics datasets, non-parametric methods (Conover, 1999; Efron and Tibshirani, 1994) are emerging as the primary methods for biomedical analysis. Non-parametric statistics have the advantage of making minimal distributional assumptions and can scale to fit the complexity of the data. A recent non-parametric robust method, *bootLong*, was developed for extracting microbial biomarkers from longitudinal microbiome data based on a moving block bootstrap approach (Jeganathan *et al.*, 2018). It accounts for within-subject dependency by using overlapping blocks of repeated observations within each subject. It then infers biomarkers based on approximately pivotal statistics. Although *bootLong* shows promising results in identifying microbial biomarkers in microbial longitudinal studies, it does not provide time intervals of differences between the study phenotypes. Another method, *MetaLonDA*, has been proposed to find time intervals of significant microbial biomarkers using a permutation test (Metwally *et al.*, 2018). *MetaLonDA* is tailored to microbial experiments through the use of a negative binomial distribution. Similarly, splinectomeR was introduced recently to test whether two groups of individuals statistically follow different trajectories over time or not through the use of LOESS regression (Shields-Cutler *et al.*, 2018). It also has the ability to identify time intervals of significant markers.

In this article, we introduce a robust method to perform longitudinal differential analysis on omics features in order to identify time intervals of differences between study groups. The method is based on a semi-parametric approach, where we use smoothing splines to model longitudinal data and infer significant time intervals of changes in omics features based on an empirical distribution constructed through a permutation procedure. The proposed method can handle all types of inconsistencies in sample collections and adjust for subjects' specific baseline. Identifying biomarkers and their significant time interval differences can inform intervention strategies (drugs, probiotics, antibiotics and supplements), and most importantly, may indicate the best time for interventions to be administered to patients. The method achieved a correctly calibrated type-I error rate and is robust to data collection inconsistencies that commonly occur in longitudinal human studies. Application of the proposed method to iPOP cohort revealed a multitude of sex differences in dynamic respiratory infection response. To our knowledge, this is the first study to investigate sexual dimorphism in infection response with frequent temporal sampling and delineation of the dynamic infection response for each sex. We also applied *OmicsLonDA* on a longitudinal lipidomics study on preeclampsia for the identification of time intervals that lipids are significantly different between pregnancy with and without preeclampsia. We provide an open-source Bioconductor R package, *OmicsLonDA* (Omics Longitudinal Differential Analysis), for widespread availability.

## 2. Materials and methods

**Fig. 1.**
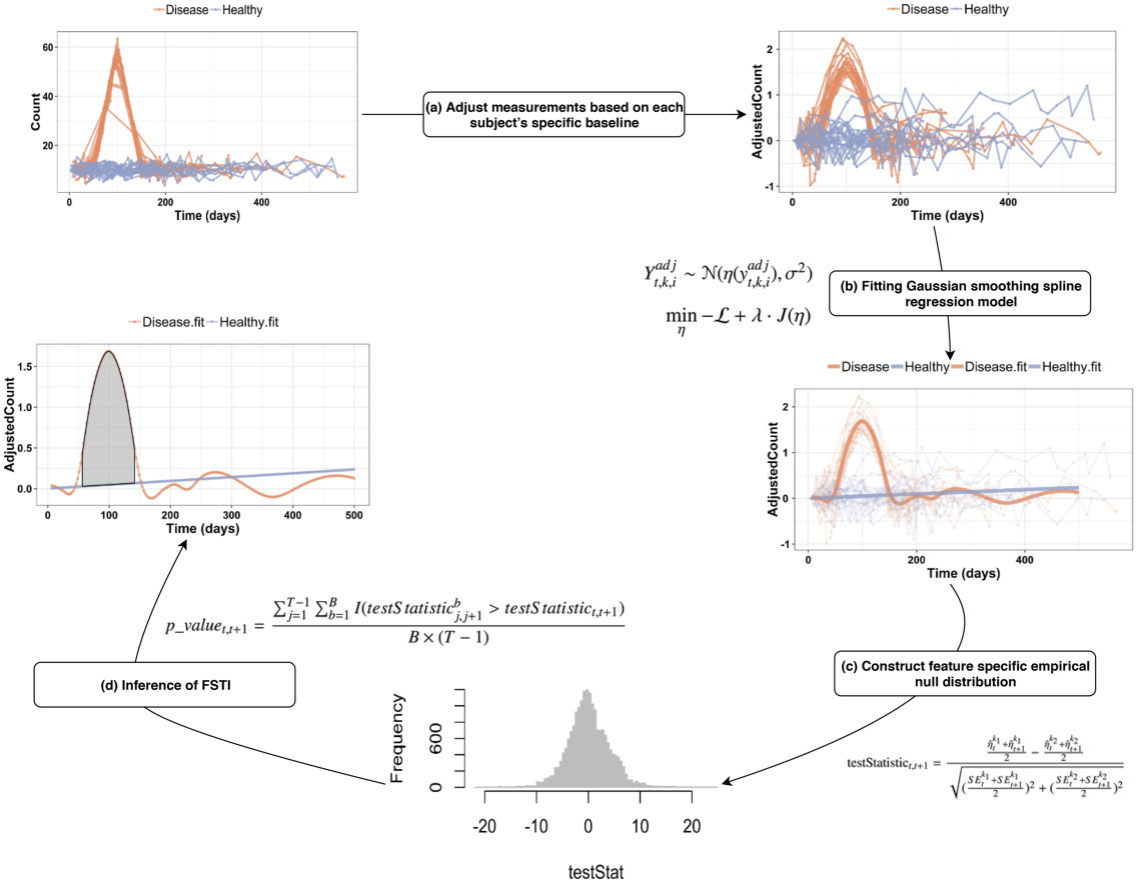
Overview of the main steps of *OmicsLonDA*: adjust measurements based on each subject’s specific baseline, fitting Gaussian smoothing spline regression model, permutation test to generate an empirical distribution of the test statistic and inference of feature’s significant time intervals (FSTI)

The proposed method, *OmicsLonDA*, aims to find the feature's significant time intervals (FSTI) of differences between each pair of the tested groups (e.g. healthy versus diseased, male versus female, etc.). The method works on unpaired experiment design, where subjects' longitudinal samples are related to only one of the tested groups. We model the longitudinal data in a time-series model using a spline kernel. Although, in theory, longitudinal data should be correlated, the first-order auto-correlation is not high [e.g. 0.19 in the longitudinal microbiome data from the Human Microbiome Project (Zhou *et al.*, 2019)]. This is mainly due to the fact that longitudinal samples are taken far apart from each other [e.g. weeks to months in Zhou *et al.* (2019)]. For this reason, we do not consider auto-correlation in our model due to the complexity of assuming a valid dependency structure. The input data to the method is the processed (filtered based on quality control thresholds, annotated, quantified, normalized, corrected for batch effect and sequencing depth) measurements of any of the omics experiments, such as genes expression from RNAseq experiments (Reuter *et al.*, 2015), proteins levels from proteomics experiments (Van Eyk and Snyder, 2018), metabolites intensities from metabolomics experiments (Kellogg *et al.*, 2018), microbial abundance from metagenomics experiments (Metwally *et al.*, 2016). The data processing output of each of these omics assays can be summarized in a matrix *C* with a dimension of *m *×* n* where *m* denotes the number of omic features and *n* denotes the number of samples. *C*(*i, j*) represents the quantity from sample *j* that is annotated to feature *i*. The proposed method is based on four main steps as shown in Figure 1: (a) adjust measurements based on each subject's profile, (b) fitting the Gaussian smoothing spline regression model, (c) permutation test to generate an empirical distribution of the test statistic of each time interval and (d) inference of significant time intervals of omics features. The details of the method are described in the following sections.

### 2.1 Adjusting for subject's personal profile

Interpersonal omics values can vary dramatically between subjects. Usually, people cluster according to themselves (Zhou *et al.*, 2019). Hence, there is a need to adjust longitudinal samples based on the subject's profile. In this work, we implemented two techniques for adjusting personal profiles. The first strategy is based on using the first sample of the study as the baseline and adjusting each following sample to the baseline. The baseline timepoint is usually chosen to be the sample prior to perturbation (e.g. infection, vaccination, surgery, etc.), or at a steady-state condition. This strategy is effective when the baseline timepoint is right before the perturbations. For each omic feature *f* under consideration, we first adjust for the difference in the personal baseline. Our strategy is to calculate the log-ratio between omic feature's level of each timepoint *t* to the level of the same omic feature at the subject's chosen baseline *t_b_* (Eq. 1), where *y_i, t_* is the measure of the omic feature of subject *i* at time point *t*, and *t_b_* is the *i*th subject's baseline. Besides adjusting for the personal baseline, the logged ratio reduces the positive skewness of the distribution while stretching out the lower end. Also, it makes the within-group variability more similar across groups, which in turn makes the homoscedasticity assumption by the following modeling acceptable. The second strategy is to use min–max scaling to normalize each feature's measurements (Eq. 2). For each feature's time series of subject *i*, the minimum value of that feature gets transformed into a 0, the maximum value gets transformed into a 1, and every other value gets transformed into a decimal between 0 and 1. This normalization step is crucial to be able to emphasize the time-series pattern rather than its amplitude, which implicitly corrects for the differing baseline measurements between subjects. However, min–max normalization is not robust in handling outlier’s measurement within any time series. Hence, outliers need to be removed as a pre-processing step prior to performing min–max normalization.

(1) $$y_{i,t}^{\mathrm{adj}}=\log\frac{y_{i,t}}{y_{i,t_{b}}},$$

 

(2) $$y_{i,t}^{\mathrm{adj}}=\frac{y_{i,t}-\min(y_{i})}{\max(y_{i})-\min(y_{i})}.$$

### 2.2 Fitting Gaussian smoothing spline regression model

For each omic feature *f = 1, …, F* from the candidate list, the data under consideration are the random variables $Y_{t,k,i}^{\mathrm{adj}}$ or their observations $y_{t,k,i}^{\mathrm{adj}}$ of level or mapped reads of the *i*th subject of group *k* to the feature *f* at timepoint *t*, where *t = 1, …, T*, *k* = 1,2, and subject *i = *1*, …, n^k^*. The random variable $Y_{t,k,i}^{\mathrm{adj}}$ is assumed to follow $Y_{t,k,i}^{\mathrm{adj}}∼N(\eta (y_{t,k,i}^{\mathrm{adj}}),\;\sigma^{2})$, where $\eta (y_{t,k,i}^{\mathrm{adj}})$ is the cubic spline function to be estimated from the data. We seek the estimation of model parameters by solving the penalized likelihood function in (Eq. 3) using a piecewise cubic polynomial minimizer. In the objective function, L=logL(η|Y) encourages the goodness of fit, $J\left(\eta \right)$ is a roughness penalty that is added to the minus log-likelihood to quantify the smoothness of η, which is essentially the inner product in a reproducing kernel Hilbert space (Gu, 2013). The λ in (Eq. 3) controls the trade-off between the goodness of fit and the smoothness of the spline and can be determined using cross-validation (Gu, 2013). For each feature, we solve (Eq. 3) for each one of the two tested groups, which leads to two smoothing splines, one for each group.

(3) $$\min_{\eta }-L+\lambda J\left(\eta \right).$$

Once we have the two smoothing splines, one that fits each group's longitudinal samples, we then calculate the test statistic for each of the *T* *−* 1 time intervals, where *T* is the number of time intervals that span the study period. We developed studentized test statistics that quantify differences between the two splines for each time interval. The formula represents the area between the two splines for each time interval (*t, t + *1) as shown in (Eq. 4), where $A_{t,t+1}^{k_{1}}$ and $A_{t,t+1}^{k_{2}}$ denote the area under the spline curve from time *t* to *t + *1 for Group 1 and Group 2, respectively, *t = *1,…*, T −* 1, and SE represents the standard error. Usually, the predicted time intervals are equidistant, as shown in Figure 1. Therefore, (Eq. 4) can be rewritten in terms of the spline function $\hat{\eta }$ as shown in (Eq. 5). Under the null hypothesis of no difference between the groups at the specific window, we expect the test statistics to take values near 0, with variance estimated using a permutation procedure described next.

(4) $${\mathrm{testStatistic}}_{t,t+1}=\frac{A_{t,t+1}^{k_{1}}-A_{t,t+1}^{k_{2}}}{\sqrt{{\left(\frac{{\mathrm{SE}}_{t}^{k_{1}}+{\mathrm{SE}}_{t+1}^{k_{1}}}{2}\right)}^{2}+{\left(\frac{{\mathrm{SE}}_{t}^{k_{2}}+{\mathrm{SE}}_{t+1}^{2}}{2}\right)}^{2}}}$$

 

(5) $${\mathrm{testStatistic}}_{t,t+1}=\frac{\frac{{\hat{\eta }}_{t}^{k_{1}}+{\hat{\eta }}_{t+1}^{k_{1}}}{2}-\frac{{\hat{\eta }}_{t}^{k_{2}}+{\hat{\eta }}_{t+1}^{k_{2}}}{2}}{\sqrt{{\left(\frac{{\mathrm{SE}}_{t}^{k_{1}}+{\mathrm{SE}}_{t+1}^{k_{1}}}{2}\right)}^{2}+{\left(\frac{{\mathrm{SE}}_{t}^{k_{2}}+{\mathrm{SE}}_{t+1}^{2}}{2}\right)}^{2}}}.$$

Under the null hypothesis of no difference between the groups at the specific window, we expect the test statistics to take values near 0, with variance estimated using a permutation procedure described next.

### 2.3 Inference of significant time intervals via permutation procedure

We perform a permutation procedure by permuting the sample group labels *k*. The permutation is done *B* times, and after each permutation, we calculate the ${\mathrm{testStatistic}}_{j,j+1}^{b}$for the null hypothesis for each time interval. Since all longitudinal samples from the same participant have the same group label after each permutation, the auto-correlation correlation is preserved across subjects, and hence, type 1 error remains the same throughout the permutation procedure. Subsequently, the pvalue of each interval of the tested feature *f* is calculated using (Eq. 6) when ${\mathrm{testStatistic}}_{t,t+1}$is positive and (Eq. 7) when it is negative, where *T* *−* 1 denotes the number of time intervals, and *I*(.) is an indicator function. The pvalue is adjusted for multiple testing using Benjamini–Hochberg (BH) to control for the false discovery rate. For each feature *f*, significant time intervals are those with ${\mathrm{pvalue}}_{t,t+1}<\alpha$, where α is the significance level.

(6) $$\begin{array}{c} {\mathrm{pvalue}}_{t,t+1}=\frac{\sum_{j=1}^{T}\sum_{b=1}^{B}I({{\mathrm{testStatistic}}_{j,j+1}^{b}>\mathrm{testStatistic}}_{t,t+1})}{B\times (T-1)},\;\\ \mathrm{where}\;b=1,\ldots ,B \end{array}$$

 

(7) $$\begin{array}{c} {\mathrm{pvalue}}_{t,t+1}=\frac{\sum_{j=1}^{T}\sum_{b=1}^{B}I({{\mathrm{testStatistic}}_{j,j+1}^{b}<\mathrm{testStatistic}}_{t,t+1})}{B\times (T-1)},\;\\ \mathrm{where}\;b=1,\ldots ,B. \end{array}$$

## 3. Results

### 3.1 Performance evaluation using simulated data

**Fig. 2.**
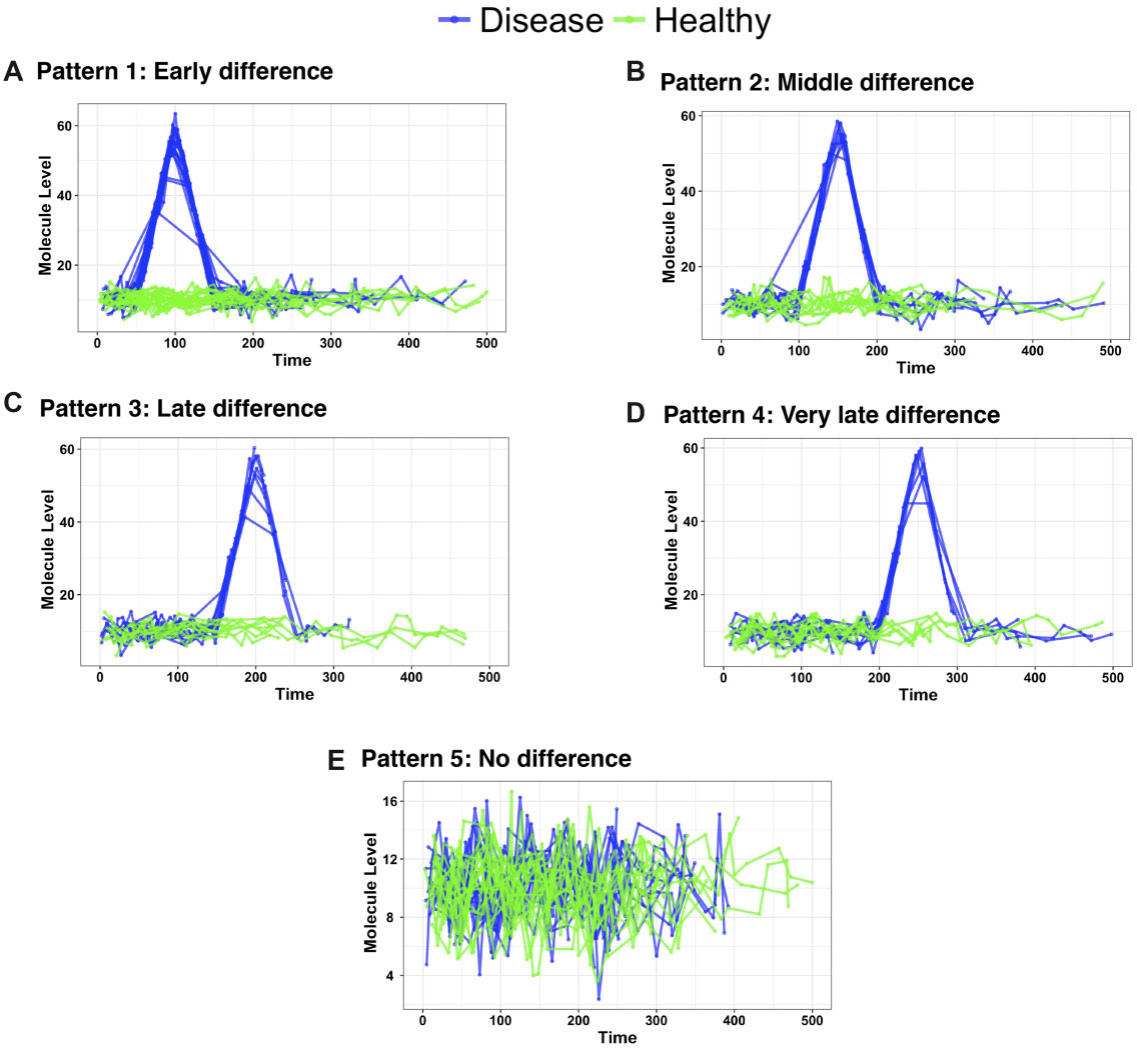
Examples of simulated features from the 5 patterns we have in this study. The first pattern indicates that the change between the two groups happened 50 days from the start of the perturbation and lasts till 150 days, Pattern 2 shows differences between 100 and 200, Pattern 3 shows differences between 150 and 250, Pattern 4 shows differences between 200 and 300, Pattern 5 has no change at all between the two groups and act as a negative control

To measure the performance of our proposed method in identifying the significant time intervals of omics features, we simulated datasets that mimic all variations in human sample collection, such as non-uniform time gap between samples, subjects have a different number of samples over the study period, a different baseline for each subject, subjects drop out late in the study and missing data. We used *SimStudy* (Goldfeld 2018) to simulate five datasets with different patterns across the study course, as shown in Figure 2. We simulated 1000 features from each pattern. The Time 0 indicates the start of the study or the start of the perturbation. In our simulations, we assumed that the covariance structure between consecutive timepoints follows first-order autoregression AR(1) with a correlation coefficient ***ρ*** = 0.4. Datasets were simulated for 10 individuals from each group. Then, to mimic variability in sample collections, we sampled data points with a variable number of subjects and a variable number of samples per subject. The generated longitudinal data have a varying number of timepoints as well as varying time intervals between each measurement period. We assumed that the number of timepoints per subject follows a truncated Poisson distribution with λ=20.

Simulated omic features of the first pattern (Fig. 2A) were simulated with a mean **μ**(*t*), which follows (Eq. 8), where ℕ denotes normal distribution, *t* = 0, …,500, and σ ∈{1,5}. The first pattern indicates that the change between the two groups happens 50 days from the start of the perturbation and lasts till 150 days, Pattern 2 (Fig. 2B) shows differences between 100 and 200, Pattern 3 (Fig. 2C) shows differences between 150 and 250, Pattern 4 (Fig. 2D) shows differences between 200 and 300 and Pattern 5 (Fig. 2E) does not have change at all between the two groups and act as a negative control. The purpose of simulating these various patterns is to benchmark the proposed method performance while there are fewer samples at the period that have differences between groups since subjects dropping out of most of the longitudinal studies is directly proportional to the time.

(8) μ(t)=N(10,σ)+t-50, 50<t≤100N(10,σ)+150-t, 100<t≤150N10,σ, otherwise.

We evaluated the performance of the *OmicsLonDA* in identifying significant time intervals from each one of the five patterns described above. In our analysis, we used B = 1000 permutations to construct the empirical distribution, a significance level of α=0.05, and adjusted for multiple testing using the BH method. We tested 500 intervals for each feature (*T* = 1, …, 501). $\mathrm{Specificity}=\frac{\mathrm{TN}}{\mathrm{TN}+\mathrm{FP}}$ and $\mathrm{Sensitivity}=\frac{\mathrm{TP}}{\mathrm{TP}+\mathrm{FN}}$, for each pattern, were measured for each feature independently, where TP is the number of significant time intervals that were correctly identified by the method, FN is the number of significant time intervals that were missed by the method, TN is the number of insignificant time intervals that were identified as insignificant, and FN is the number of significant time intervals that were identified as insignificant. Then, average specificity and sensitivity were measured among the 1000 features for each pattern. We benchmarked two variants of *OmicsLonDA* based on the model they use to fit the longitudinal data for each group; (i) OmicsLonDA with smoothing spline ANOVA (*OmicsLonDA_SSANOVA*) and (ii) *OmicsLonDA* with Gaussian additive mixed models (*OmicsLonDA_GAMM*). GAMM allows fitting smoothing terms to model time-series data, and it uses subject ID as a random effect. No covariates were added in this simulation study. We also benchmarked *OmicsLonDA* against *MetaLonDA* and *splinectomeR*. We choose to benchmark against *MetaLonDA* and *splinectomeR* since, to our knowledge, they are the two methods that were developed mainly to identify time interval of significance between the two tested groups. There are three key differences between *OmicsLonDA* and *MetaLonDA*: (i) *MetaLonDA* does not correct for personal baseline, (ii) *MetaLonDA* uses negative binomial smoothing spline when used with microbiome data and LOESS regression otherwise and (iii) *MetaLonDA* uses a different formula for testStatistic that only include the area between the curves of the two groups without adjusting of the standard error in their estimation. In our benchmarking experiments, we ran *MetaLonda* with LOESS regression and 1000 permutations, and all other parameters were left as default. For *splinectomeR*, we ran the benchmarking evaluation with cut_low = 0 inorder not to filter out any subjects based on small number of time points, and the rest of parameters were left as default.

Table 1 demonstrates the high level of specificity of *OmicsLonDA* (>0.99) among all five tested patterns, with *OmicsLonDA_GAMM* has slightly more specificity over the first four patterns, and *OmicsLonDA_SSANOVA* has slightly more specificity in Pattern 5. On the other hand, *MetaLonDA’s* specificity is ∼0.80 among all first four patterns, and 0.97 in Pattern 5. splinectomeR followed similar trends to *MetaLonDA, high sensitivity and low specificity.*  Table 1 shows the sensitivity of all benchmarked methods for all patterns, except Pattern 5. This is because all features in Pattern 5 were simulated to not have any significant differences in the time intervals between the two compared groups. *splinectomeR* has the highest sensitivity (∼1) among the compared methods. This high sensitivity can also be seen as a trade-off with the low specificity of *splinectomeR*. In general, there is a decrease in sensitivity for all methods from Pattern 1 to Pattern 4. This decrease in sensitivity is expected due to the fact that as the time intervals that are significantly different between the two groups shift to the right (later in the study course, which was implemented in our simulations), there are more participants dropping out of the study, and hence there is lower power of each method to detect the significantly differential time intervals. *OmicsLonDA_SSANOVA* maintains reasonably high sensitivity across all patterns (Pattern 1: 0.98, Pattern 1: 0.92, Pattern 3: 0.90 and Pattern 4: 0.87). *OmicsLonDA_GAMM* has a similar sensitivity pattern to *OmicsLonDA_SSANOVA*, but surprisingly, the sensitivity drops significantly at Pattern 4 (0.72). These results demonstrate that *OmicsLonDA_SSANOVA* is a better choice than *OmicsLonDA_GAMM* when few samples cover the tested time interval. Additionally, Table 1 shows that low variance in simulated data (σ =1 versus 5 in Eq. 8) increases the sensitivity and specificity of all methods.

**Table 1.** Performance evaluation of identifying significant time intervals from simulated features

		*σ* = 5		*σ* = 1	
		Sensitivity	Specificity	Sensitivity	Specificity
Pattern_1 (early change)	*OmicsLonDA (SSANOVA)*	0.976	0.998	0.982	0.993
	*OmicsLonDA (GAMM)*	0.945	0.991	0.965	0.990
	*MetaLonDA*	0.993	0.833	0.992	0.881
	*splinectomeR*	1	0.698	1	0.735
Pattern_2 (middle change)	*OmicsLonDA (SSANOVA)*	0.924	0.999	0.952	0.998
	*OmicsLonDA (GAMM)*	0.948	0.995	0.963	0.997
	*MetaLonDA*	0.991	0.818	0.991	0.818
	*splinectomeR*	0.999	0.682	1	0.699
Pattern_3 (late change)	*OmicsLonDA (SSANOVA)*	0.906	0.999	0.921	0.999
	*OmicsLonDA (GAMM)*	0.934	0.995	0.957	0.998
	*MetaLonDA*	0.984	0.805	0.991	0.876
	*splinectomeR*	0.965	0.804	1	0.772
Pattern_4 (very late change)	*OmicsLonDA (SSANOVA)*	0.870	0.998	0.902	0.999
	*OmicsLonDA (GAMM)*	0.725	0.999	0.774	0.999
	*MetaLonDA*	0.926	0.795	0.932	0.829
	*splinectomeR*	0.995	0.729	1	0.765
Pattern_5 (no change)	*OmicsLonDA (SSANOVA)*	—	0.982	—	0.983
	*OmicsLonDA (GAMM)*	—	0.989	—	0.994
	*MetaLonDA*	—	0.972	—	0.981
	*splinectomeR*	—	0.959	—	0.962

Additionally, we evaluated the two implemented personal baseline adjusting methods (log-ratio and min–max normalization) on *OmicsLonDA* performance. Table 2 shows the sensitivity and specificity of *OmicsLonDA* when running on the five simulated patterns after each of the baseline adjusting methods. While log-ratio and min–max baseline adjusting methods have yielded a similar effect on *OmicsLonDA* specificity, min–max has yielded higher sensitivity.

**Table 2.** Evaluation of adjusting subject’s profile

	*OmicsLonDA* (log-ratio)		*OmicsLonDA* (min–max)	
	Sensitivity	Specificity	Sensitivity	Specificity
Pattern_1 (early change)	0.97	0.96	0.97	0.99
Pattern_2 (middle change)	0.93	0.96	0.93	0.99
Pattern_3 (late change)	0.88	0.98	0.9	0.99
Pattern_4 (very late change)	0.81	0.99	0.88	0.99
Pattern_5 (no change)	—	0.98	—	0.99

### 3.2 Time and memory evaluation

The running time of *OmicsLonDA* depends primarily on the number of permutations used to construct the empirical distribution for each feature. Additionally, *OmicsLonDA* can be run in parallel on multi-core platforms. However, we used one thread in our time evaluation. In our analysis of the simulated data with 1000 permutations, for each feature, *OmicsLonDA* analysis took, on average, 43 min and 47 s. The evaluation was conducted on a MAC machine with a 2.5 GHz Intel Core i7 processor and 16 GB 1600 MHz RAM.

### 3.3 Application of *OmicsLonDA* on real-world datasets

#### 3.3.1 Ipop infection multi-omics cohort

As an application for our proposed method, we used the integrative Personal Omics Profiling (iPOP) cohort, a longitudinal cohort that aims to characterize the complex host–microbial interactions in type 2 diabetes mellitus (T2DM) (Zhou *et al.*, 2019). The iPOP cohort was established to better understand T2DM at its earliest stages, where healthy or prediabetic individuals are sampled over ∼4 years in a deep multi-omics profiling of transcriptomes, metabolomes, proteomes and cytokines, as well as gut and nasal microbiome. In a total of 1091 visits, 105 participants (25–75 years old, body mass index of 19–41 kg/m^2^, 55 females and 50 males) were profiled during healthy periods and extensively during periods of respiratory viral infection (RVI), immunization and other situations that perturb human host–microbial physiology.

**Fig. 3.**
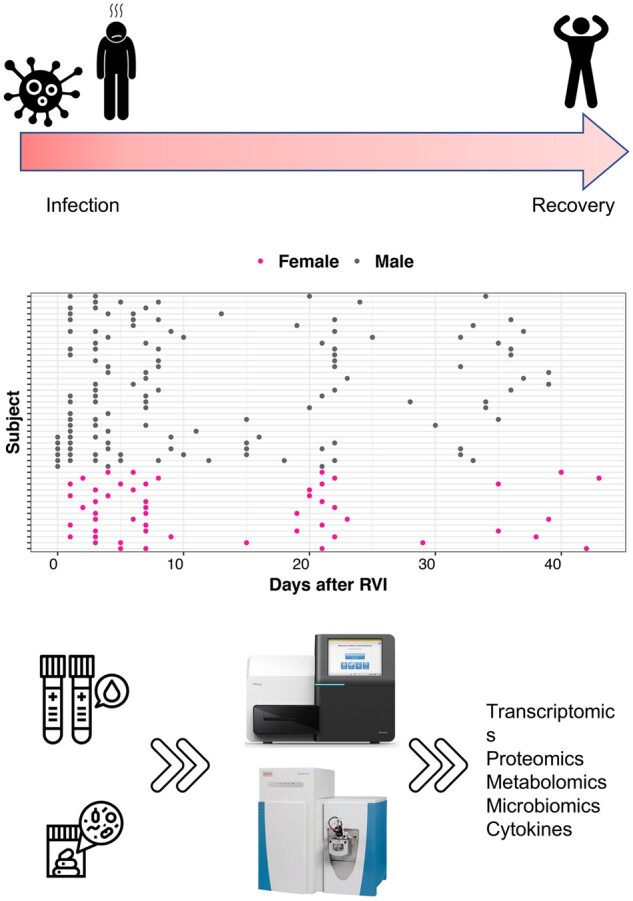
Study design of the iPOP infection cohort. Time points distributions of 44 infection episodes whose corresponding subject has at least three timepoints within 40 days following and infection incidence. Total of 180 samples from 25 subjects (12 male and 13 female). Timeline annotation of RVI episodes, where Day 0 is the first day of infection

We leveraged the power of the longitudinal multi-omics nature of the iPOP study to reveal sexual dimorphism at the molecular level following RVI episodes. Sex is considered to be an important epidemiological factor that can determine the risk for some diseases. However, the sex-dependent responses to RVIs are not well explored, especially in a multi-omics and microbiological fashion. Most of the previous studies were based on epidemiological strategy and reported the prevalence of RVI in different sex (Channappanavar *et al.*, 2017; Chiarella *et al.*, 2017; Granados *et al.*, 2017; Wang *et al.*, 2016). In this work, we utilized *OmicsLonDA* to identify longitudinal transcriptomic, metabolomic, cytokines and microbial changes between females and males following RVI. In the context of this work, we included 25 (12 male and 13 female) participants who were followed before and after RVI (44 episodes of RVI in a total of 180 RVI visits; Fig. 3). We selected episodes that have at least three samples during the first 39 days after RVI. We first adjusted each feature using min–max normalization (Eq. 2). Each feature (gene, protein, metabolite, cytokine or microbe) was tested independently. Time interval inference is based on an empirical distribution that is built for all intervals of the same feature as described previously. We tested 38 intervals (between Day 1 and Day 39). For proteins, metabolites, cytokines and microbes, we tested all quantified features from the iPOP cohort. In our analysis, we used *B* = 1000 permutations to construct the empirical distribution, significance level α=0.05, and adjusted for multiple testing using BH method.

**Fig. 4.**
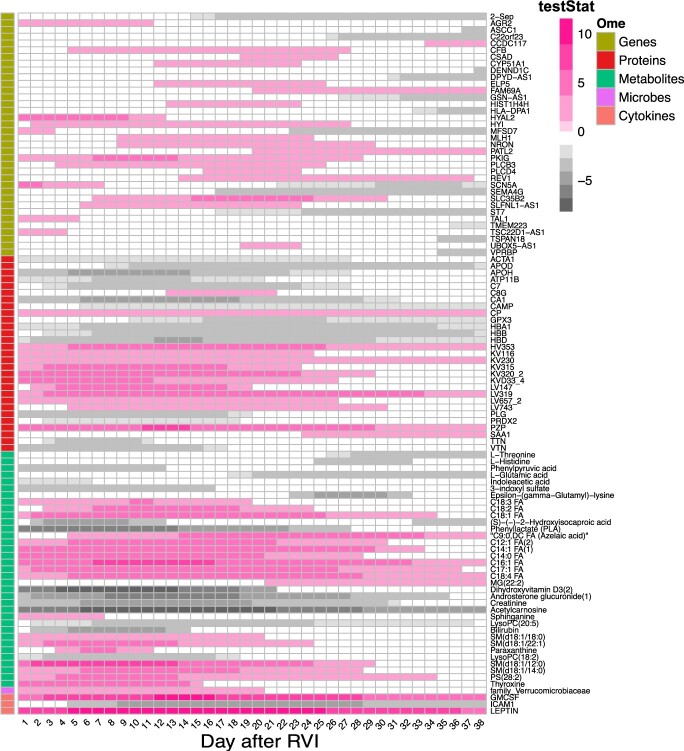
Significant time intervals of features that show differences between males and females following RVI. Each row represents a feature. Pink shaded cells indicate the corresponding feature is over-expressed in the female group, while the gray cells indicate the corresponding feature is over-expressed in the male group (A color version of this figure appears in the online version of this article.)

In total, 104 features (36 genes, 29 proteins, 35 metabolites, 3 cytokines and 1 microbe) exhibit temporal differences between males and females following RVI. Figure 4 shows a timeline summary of omic features that show the difference between males and females after RVI episodes (Supplementary Table S1). The results reveal that females were more responsive to RVI with 58 omic features being overexpressed, while 44 features were over-expressed in males, and 2 genes (MFSD7 and SCN5A) flipped their over-expression trajectory during the course of the infection episode. Females have stronger antibody responses IGLV3-19 (LV319) and IGHV3-53 (HV353). Females have a stronger adaptive response early than males, while males have more innate responses than females with increased complement proteins and increased red blood cells (HBA1, HBB, HBD). Interestingly, males have over-expression of vitamin D3 (dihydroxyvitamin) during the early infection period (Day 1 to Day 21). Females have higher leptin during the whole course of infection (1–39 days).

#### 3.3.2 Preeclampsia lipidomics cohort

We applied *OmicsLonDA* on a longitudinal lipidomics study on preeclampsia (Maric *et al.*, 2021) as a case study to demonstrate the value of *OmicsLonDA* for the identification of time intervals that lipids are significantly different between pregnancy with and without preeclampsia. Preeclampsia is a serious pregnancy complication affecting 5–10% of pregnant women, accounting for approximately 40% of fetal deaths worldwide. It not only harms maternal health but also inhibits fetal growth and causes babies to be born with immature development. Therefore, detection of preeclampsia biomarkers at early gestational age and identification of the time intervals with dramatic lipid changes in preeclampsia is crucial for preeclampsia early diagnosis and treatment.

**Fig. 5.**
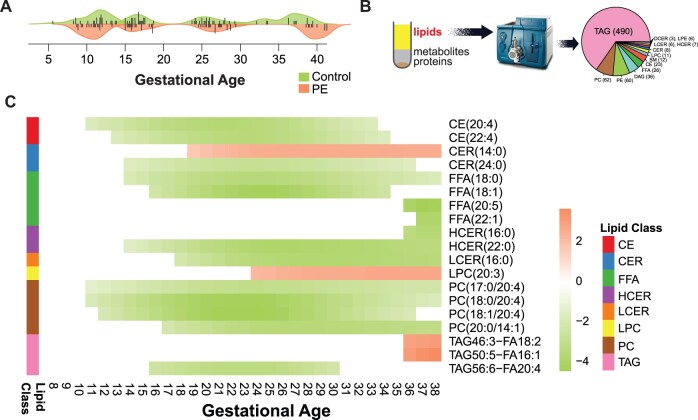
A case study of the longitudinal lipidomics data on the preeclampsia cohort. (**A**) The gestational age distribution of the collected plasma samples from the control and preeclampsia (PE) groups in each trimester in this study. (**B**) Lipids from the plasma samples were extracted and measured by the target lipidomics profiling platform Lipidyzer for 750 different lipids. (**C**) The 19 lipid species exhibit significantly different profiles between the control and preeclampsia groups by applying *OmicsLonDA*

In this longitudinal prospective study, the cohort was previously described (Maric *et al.*, 2021) with 27 and 20 women with and without preeclampsia, respectively. The plasma samples were collected from each subject at two or three-time points during pregnancy. The gestational age distribution of the preeclampsia and control groups in each trimester is shown in Figure 5A. For each plasma sample, we conducted target lipidomics analysis by applying the Lipidyzer platform for 750 lipid species composed of 13 diverse lipid classes (Figure 5B).

Following *OmicsLonDA* analysis workflow, we first adjusted the levels of each lipid at later time points using min–max normalization method (Eq.2) and normalized them to baseline. The *OmicsLonDA* test was conducted for each lipid independently. We set 1 week as one time interval unit and tested on 30 time intervals (Week 8–38). In our analysis, we used 1000 times permutations to construct the empirical distribution for each lipid. All the results were adjusted for multiple testing using BH method with a significance level α=0.05.

We identified 19 lipid species, accounting for eight lipid classes, with significant temporal differences between preeclampsia and control groups during pregnancy. Figure 5C demonstrates the time intervals of these 19 lipids that have significantly different profiles between preeclampsia and control groups (Supplementary Table S2). Interestingly, we found that most of the significant lipids belonging to the same lipid classes exhibit the same changing trends, indicating homogeneity of chemical properties and potential biological roles of lipid species from the same classes. There are two exceptions: one is CER (14:0) and CER (24:0), which may be due to the different lengths of fatty acid chains in these two ceramides. Another interesting exception is TAG 46:3 (FA 18:2), TAG 50:5 (FA16:1) and TAG 56:6 (FA20:4), showing the higher levels of the first two triglycerides in the preeclampsia group at later gestational age compared to the control group. In contrast, TAG 56:6 (FA20:4) displays the increased abundance at early pregnancy in control subjects. Further experiments are needed to investigate the explicit reasons.

## 4. Discussion

In this work, we have developed a statistical method that provides robust identification of time intervals where omics features are significantly different between groups in longitudinal multi-omics. The method is able to simultaneously identify time intervals and differential signatures by analyzing each feature separately, but across all patients. The proposed method is based on a semi-parametric approach, where using smoothing splines to model longitudinal data and infer significant time intervals of omics features based on an empirical distribution constructed through the permutation procedure. A critical need in longitudinal omics is for robust frameworks that incorporate the time dimension in statistical significance analysis. Our method was evaluated through extensive simulations (five patterns). The performance evaluation demonstrated that *OmicslonDA* has achieved a correctly calibrated type I error rate and is robust to data collection inconsistencies that commonly occur in longitudinal human studies. Moreover, the sensitivity is high in Pattern 1 and then declines slightly through Pattern 4. This decrease in sensitivity can be explained due to the decreasing number of samples collected towards the end of the study (i.e. patient dropout).

We further applied *OmicsLonDA* on two real-world datasets: (i) the iPOP longitudinal omics study for investigating sexual dimorphism on molecular response following RVI and (ii) preeclampsia cohort to identify time intervals that lipids are significantly different between pregnancy with and without preeclampsia. Recently, *OmicsLonDA* has been utilized to identify the seasonal time intervals of differentially abundant/expressed omics features between insulin-resistant and insulin-sensitive individuals (Sailani *et al.*, 2020).

Sex differences in response to infection are known (Casimir *et al.*, 2013). For both viral and bacterial infections, males are more susceptible than females, while females produce a more vigorous inflammatory response (Casimir *et al.*, 2013). Sexual dimorphism in infection response likely arises from differences in hormone status, with both testosterone and estrogen shown to modulate infection and inflammatory processes (Chrousos, 2010). Our study further adds evidence that sexual dimorphism may contribute to stages of inflammatory responses (Klein and Flanagan, 2016), with females having a stronger adaptive response early but less innate responses than male. Our analysis is the first to our knowledge that revealed a multitude of sex differences in RSV infection response with frequent temporal sampling and delineation of the dynamic infection response for each sex.

Preeclampsia is a potentially life-threatening complication during pregnancy identified by increased blood pressure and proteinuria. It is one of the leading causes of maternal and perinatal mortality and morbidity (Ghulmiyyah and Sibai, 2012). Substantial efforts have been made to detect molecular changes of preeclampsia during pregnancy at gene, protein and metabolite levels (He *et al.*, 2020; Lapaire *et al.*, 2012; Nobakht M. Gh, 2018). Nowadays, lipids are growingly recognized as key players involved in pathophysiology of preeclampsia (Anand *et al.*, 2016; Wojcik-Baszko *et al.*, 2018). For instance, arachidonic acid and its downstream products were reported to be significantly changed in preeclampsia (Balazy, 2004). Oxidized lipid species were also selected as biomarkers of preeclampsia which are related to increased reactive oxygen species (Butterfield and Lauderback, 2002). However, most of these studies mainly focused on single timepoint instead of monitoring dynamic molecular changes with multiple timepoints during pregnancy. Herein, in this study, we applied the developed *OmicsLonDA* on a longitudinal lipidomics dataset to compare lipid levels in women with and without preeclampsia. We successfully identified 19 distinct lipid species that are significantly different in preeclampsia pregnancy compared to normal pregnancy with different time intervals. Interestingly, most of the significant lipids that belong to the same lipid classes exhibit the same changing trends, indicating potentially similar biological functions these lipids may exert in preeclampsia progression. Importantly, by *OmicsLonDA*, we detected several lipids that harbored significantly different time intervals at early pregnancy phase (i.e. the first trimester) such as CE (20:4), PC (17:0/20:4), PC (18:0/20:4) and PC (18:1/20:4), which may serve as clinically meaningful biomarkers for preeclampsia early diagnosis. Intriguingly, all of these four lipid biomarkers share the same fatty acid chain arachidonic acid (fatty acid 20:4). Arachidonic acid is a polyunsaturated fatty acid-containing 20 carbons and four double bonds with a final double bond in the ω − 6 position. It is well-documented that arachidonic acid and its products eicosanoids play important roles in inflammatory processes (Higgins and Lees, 1984). They have been reported as biomarkers of preeclampsia previously (Balazy, 2004). Our results not only support the previous findings but also revealed more potentially involved lipid biomarkers by leveraging the advantages of the longitudinal data as well as the merit of *OmicLonDA*.

There are two factors that need to be considered before applying *OmicsLonDA* to any longitudinal dataset: (i) baseline adjustment method and (ii) number of permutations. Firstly, if subjects have samples right before perturbation, baseline adjustment based on log-ratio (Eq. 1) would be preferred since the adjusted time series would reflect the perturbation effect for each person. However, obtaining a sample right before perturbation may not be accessible in many longitudinal multi-omics studies. Hence, adjusting based on min–max (Eq. 2) would be a more feasible solution. The downside of using the min–max method is that the adjusted time series may not be following normal distribution, violating the assumption of Gaussian smoothing spline. This may not be a concern for *OmicsLonDA* since the identification of significant time intervals is based on a non-parametric permutation test. Secondly, the running time of *OmicsLonDA* depends primarily on the number of permutations. In our simulations and real-world datasets, we used 1000 permutations to ensure stable null distribution construction. However, we have seen that a similar stable null distribution can be achieved with 100 permutations, which can be used as a fast approach to identify time intervals. However, we do not recommend running *OmicsLonDA* with less than 100 permutations since the results may not be consistent between different runs.

*OmicsLonDA* elucidates not only differentially regulated molecules but indicates the temporal window over which the differential regulation occurs to provide a nuanced and detailed understanding of biological dynamics. In the future, we plan to utilize the identified multi-omics features and their significant time intervals through non-parametric Bayesian dynamic networks to infer the causality of phenotypes based on a phased correlation between features' time intervals and phenotype onset. Another avenue for improving the proposed method is to incorporate auto-correlation between longitudinal samples into the model fitting. Also, in the proposed method, time intervals to be tested are a user-defined parameter. In the future, we plan to develop a learning method that selects non-trivial intervals that span several timepoints. *OmicsLonDA* is publicly available on the Bioconductor repository (https://bioconductor.org/packages/OmicsLonDA).

## Supplementary Material

btac403_Supplementary_Data
